# Profiles of Mathematics Anxiety Among 15-Year-Old Students: A Cross-Cultural Study Using Multi-Group Latent Profile Analysis

**DOI:** 10.3389/fpsyg.2019.01217

**Published:** 2019-05-28

**Authors:** Xizhen Fan, Ronald K. Hambleton, Minqiang Zhang

**Affiliations:** ^1^School of Psychology, South China Normal University, Guangzhou, China; ^2^College of Education, University of Massachusetts Amherst, Amherst, MA, United States; ^3^Center for Studies of Psychological Application, South China Normal University, Guangzhou, China; ^4^Guangdong Key Laboratory of Mental Health and Cognitive Science, South China Normal University, Guangzhou, China

**Keywords:** mathematics anxiety, multi-group latent profile analysis, cross-culture, cultural differences, mathematics anxiety profiles, mathematics performance

## Abstract

Using PISA 2012 data, the present study explored profiles of mathematics anxiety (MA) among 15-year old students from Finland, Korea, and the United States to determine the similarities and differences of MA across the three national samples by applying a multi-group latent profile analysis (LPA). The major findings were that (a) three MA profiles were found in all three national samples, i.e., Low MA, Mid MA, and High MA profile, and (b) the percentages of students classified into each of the three MA profiles differed across the Finnish, Korean, and American samples, with United States having the highest prevalence of High MA, and Finland the lowest. Multi-group LPA also provided clear and useful latent profile separation. The High MA profile demonstrated significant poorer mathematics performance and lower mathematics interest, self-efficacy, and self-concept than the Mid and Low MA profiles. Same differences appeared between the Mid and Low MA profiles. The implications of the findings seem clear: (1) it is possible that there is some relative level of universality in MA among 15-year old students which is independent of cultural context; and (2) multi-group LPA could be a useful analytic tool for research on the study of classification and cultural differences of MA.

## Introduction

With the arrival of the big data era, mathematical proficiency is becoming increasingly important for students’ schooling and workers’ occupation (Dowker et al., 2016), especially critical for the science, technology, engineering, and mathematics (STEM) workforce (Foley et al., 2017). The demand for mathematical skills is predicted to increase and many countries are making an effort to improve their mathematics education (Lacey and Wright, 2009). While attention is increasingly being devoted to mathematics instruction quality, a critical influential factor, mathematics anxiety (MA), is often ignored (Foley et al., 2017). As evidence showed that mathematics is one of the most difficult subjects for students to learn; and some students have a mathematical learning disability (e.g., Lewis and Fisher, 2016). But not all of these learning difficulties result from cognitive factors, emotional factors, such as MA, also play a large role in mathematics learning (Aiken, 1970; Ashcraft and Kirk, 2001). Therefore, more efforts should be made to obtain a better understanding of MA and explore effective ways to alleviate its negative impact on students’ mathematics learning.

According to Richardson and Suinn, MA can be defined as “feelings of tension and anxiety that interfere with the manipulation of numbers and the solving of mathematical problems in a wide variety of ordinary life and academic situations” (Richardson and Suinn, 1972, p. 551). It involves negative cognitions, avoidance behaviors, feelings of pressure and performance inadequacy (Shahram and Farahman, 2011). People may experience MA in formal settings, when taking mathematics tests, or in everyday settings, like calculating tips at a restaurant after meals (Ashcraft and Moore, 2009).

As a significant affective emotional factor, MA may lead to avoidance of mathematics behaviors and damage the working memory resources needed for difficult mathematics problems solving at the moment (Ganley and Vasilyeva, 2014), which can impede both learning and performance in mathematics, hence will bring deleterious effects on educational and occupational or even overall life outcomes (Betz, 1978; Trice and Ogden, 1987). MA sufferers also demonstrate decreased mathematics self-confidence, enjoy mathematics less (Hembree, 1990). Research has shown that MA correlates −0.34 and −0.31 with mathematics test scores in precollege samples and college samples respectively; −0.30 and −0.27 with high school and college mathematics grades respectively (Ashcraft and Kirk, 2001). The Program for International Student Assessment (PISA) data also showed that in 63 of the 64 participating education systems in PISA 2012, students reporting higher MA displayed lower mathematics performance than those with lower levels of MA (OECD, 2013). According to a meta-analysis by Hembree (1990), correlations between MA and non-cognitive mathematics learning outcomes were uniformly negative and frequently quite strong (Hembree, 1990): MA correlated −0.73 with mathematics interest, and −0.82 with mathematics self-efficacy in school pupils. Among college students, the mean correlations were −0.47 between MA and mathematics interest, and −0.65 between MA and mathematics self-efficacy. Ma and Kishor (1997) also did a meta-analysis by examining 26 studies on the relationship between MA and mathematics performance among elementary and secondary students, and it showed a significant negative correlation of the common population for the relationship (*r* = 0.27). Most research also found that MA correlates negatively to mathematics self-concept (Hembree, 1990; Jain and Dowson, 2009; Goetz et al., 2010; Hoffman, 2010). While it is a common agreement that MA correlates negatively to mathematics achievement and non-cognitive learning outcomes, it remains unknown whether poor mathematics performance causes MA or MA reduces mathematics performance. Some researchers suggested that there might be a bidirectional relationship between MA and mathematics achievement, in which the two affect each other in a vicious cycle (Carey et al., 2016).

As an impediment to mathematical development, MA appears among students as early as in Grade 2 (Sorvo et al., 2017), even in countries with low MA level, such as Finland (Lee, 2009). Research showed that about 11% of college students showed high MA and may need professional counseling (Richardson and Suinn, 1972); about 68% of students taking mathematics classes experienced high MA (Betz, 1978); and around two-thirds of American adults fear and hate mathematics (Burns, 1998). Chinn (2009) suggested that 2 to 6% of secondary school pupils in England showed high MA. The Organization for Economic Co-operational Development (OECD) also reported that the average number of adolescents who feel very nervous about mathematics problems solving reached 30.6% (OECD, 2015). As can be seen, numbers of the prevalence of MA in the above-mentioned studies are quite different, and are probably to be relied on the samples, on the instruments applied to measure MA, and on the criteria used to categorize people as “mathematics anxious” in these studies (Dowker et al., 2016).

Differences in the prevalence of MA in different studies may reveal that different countries have not only different levels of actual mathematics performance but also different levels of MA, just as PISA results showed (OECD, 2013). Students in high-achieving countries, such as Finland, could be low in MA because they are good at mathematics; on the other hand, they could have high level of MA, because high importance is often attached to mathematics performance in such countries, which makes failure in mathematics much more threatening (Dowker et al., 2016). However, Lee (2009) compared the universals and specifics of MA across 41 countries participated in PISA 2003 and indicated that the relation of the average score of MA among students in a country to that country’s overall level of mathematics performance was not consistent: in some high-achieving European countries, such as Finland, students tended to show low level of MA; while in some high-achieving Asian countries, such as Korea, students tended to show high level of MA (Dowker et al., 2016).

The inconsistency of previous studies may result from the using of continuous or sum of MA scores. Up to date, most measures of MA result in continuous scores, and we don’t have a clear criterion for students to be categorized as high in MA (Dowker et al., 2016). The challenge of using continuous or sum scores to determine the level of MA would be how and where to draw the dividing line to capture the real quantitative difference. Different dividing lines may result in various figures of how many individuals being categorized as high or low in MA. In addition, using continuous or sum scores focused on capturing information about MA for the overall sample might obscure some important differences among different groups of the overall sample (Rosato and Baer, 2012). Such a limitation can be overcome by applying latent variable mixture models which are useful for identifying classes of latent variables (Yang, 2006).

The latent variable mixture models, such as latent profile analysis (LPA), are widely used to identify profiles which have as little variation within a profile and as much variation between different profiles as is possible (McMullen et al., 2018). The objective of LPA is the same as with cluster analysis, but LPA identifies cases using a model-based method (Muthén and Muthén, 2000; Vermunt and Magidson, 2002) and can yield more reliable results than cluster analysis. Besides, multigroup LPA (MLPA) allows for direct comparison of different response patterns on latent variables across samples (Geiser et al., 2006), thus can give more information of the differences of MA in different contexts.

On the other hand, most of the previous studies on MA were carried out in a single western context by using continuous sum scores of MA, over half of which were in North America, mostly in the United States (Morsanyi et al., 2016). This may be a critical limitation in the literature because results may not generalize across cultural contexts. Investigation of MA in a single context was only a partial picture, and might not permit direct comparisons of MA in different regions of the world, thus limits the understanding of MA and its cultural differences. Besides, while many countries around the world are trying to improve their mathematics education, research has been carried out to understand why some countries showed higher achievement in mathematics than others at the same time (Mullis et al., 2012; Shimizu and Kaur, 2013). A critical step for research on MA is to investigate it in a cross-cultural context and explore the potential similarity and difference of MA among different countries.

This study employs LPA and MLPA to explore subtypes of MA among 15-year old students and the cultural differences by using samples from three different countries.

So far, no specific research into the classification of MA has been conducted, but some pilot studies in test anxiety have been done, one of them by von der Embse et al. (2014). Results of von der Embse et al.’s (2014) study indicated three different test anxiety categories (i.e., high, mid, and low test anxiety category). Research has shown that there are substantial correlations (*r* = 0.65–0.75) between test anxiety and MA (Bruck, 1981; Chiu and Henry, 1990). Richardson and Woolfolk (1980) argued that “mathematics anxiety can be viewed as a form of test anxiety” (p. 271). Theoretically, there should be a “nesting effect” for MA and test anxiety. Thus, on the basis of von der Embse et al. ’s previous finding, it’s hypothesized an optimal solution of three MA profiles could emerge in the current study, consisting of individuals who showed low, mid, or high level of MA.

As for the cultural difference of MA, there has been no specific research on the study of this topic either. Would the hypothesis of three MA profiles be consistent across different cultural contexts? Would there be any differences in the latent profile pattern of MA among different cultural contexts?

The objectives of the current study are to investigate: (a) whether there are distinct unique profiles of MA among 15-year old students; (b) whether the latent profile pattern of MA is the same among three different countries. We intended to explore some more specific information of MA besides the information of the overall level and investigate potential differences of MA among different countries to obtain a better and deeper understanding of MA, which might be able to shed light on the alleviation of MA and improve mathematics education worldwide.

## Materials and Methods

### Data

PISA 2012 mathematics assessment and survey questionnaire were used for this study. The PISA 2012 project, conducted by OECD, released the public-use data on both students’ mathematics test results and responses on the background questionnaires (OECD, 2013). There are 65 countries participated in PISA 2012. However, to make this study less complex and doable, only three of them, Finland (FIN), Korea (KOR)^1^ and the United States (USA), were chosen to represent the cross-cultural context for this study. This was based on three considerations: (a) Finland, Korea, and the United States are from three different cultural areas (Finland the European culture circle, Korea the Asian culture circle, United States the North American circle), they are culturally different (Kroeber, 1931); (b) according to the results from PISA 2012, they showed quite different levels in mathematics performance, which can be used to explore the relationship between MA and mathematics performance; (c) previous studies showed that relationship between overall mathematics performance and the average MA level of these three countries was not consistent: Korea demonstrated high mathematic anxiety and mathematics performance, while Finland showed high mathematics achievement and low MA, the United States showed mid MA and mathematics performance (e.g., Lee, 2009). These results can be considered as an important reference and be compared with the results of the current study.

Analyses in this study drew on the samples of 8,829 Finnish students, 5,033 Korean students, and 4978 American students who have taken the PISA 2012 mathematics assessment. It should be noted that as PISA 2012 used a rotated design to collect data which resulted in each item being administered to approximately two-thirds of students from the entire sample, some analyses used subsets of the samples. The final student weight (W_FSTUWT) was used to obtain generalizable estimates for the target population.

### Variables

#### Mathematics Anxiety

Five items, which describe different aspects of MA, were used with a four-point Likert scale from the PISA 2012 project. The five items were: (a1) I often worry that it will be difficult for me in mathematics classes; (a2) I get very tense when I have to do mathematics homework; (a3) I get very nervous doing mathematics problems; (a4) I feel helpless when doing a mathematics problem; (a5) I worry that I will get poor grades in mathematics, with four response categories: “1 strongly agree,” “2 agree,” “3 disagree,” and “4 strongly disagree” (OECD, 2014). All five items were reversely recoded as 4-0, 3-1, 2-2, 1-3, so higher scores corresponded to higher levels of MA.

#### Mathematics Learning Outcomes

Variables of students’ mathematics learning outcomes, which included mathematics performance and non-cognitive outcomes, were included in this study for comparisons of characteristics across different MA profiles to validate the classification. The objective of LPA is to assign students into categories which have as little variation within a category and as much variation between categories as is possible (McMullen et al., 2018). If the classification is accurate, there should be significant differences concerning mathematics performance and non-cognitive outcomes between MA profiles, since MA is an important predictor of mathematics learning and negatively correlates to mathematics learning outcomes, i.e., higher MA leads to lower achievements in mathematics learning (e.g., Ashcraft and Kirk, 2001).

Mathematics performance was measured by the PISA 2012 mathematics assessment, and five plausible values for each student were produced and coded PV1MATH to PV5MATH. Plausible values are computed by randomly drawing numbers from posterior distributions of ability or scores (Holland and Hoskens, 2003), they are not actural mathematics scores of each student and should not be used as such in the PISA dataset (OECD, 2014). Therefore, analysis of plausible values for mathematics performance in this study was undertaken five times, one time with each one of the five plausible values, and then the results were averaged.

Mathematics interest (INTMAT), mathematics self-efficacy (MATHEFF), and mathematics self-concept (SCMAT) were chosen as students’ non-cognitive outcomes of mathematics learning. Different items with the same four-point Likert scale were used for these three variables. For each of the variables, weighted likelihood estimation (WLE) was used and transformed to an international metric to obtain individual participant scores (Warm, 1989). Higher scores corresponded to higher level performance on these variables.

All alpha reliabilities for the five scales (or variables) across the three samples in this study were in access of 0.70, and 14 of them exceeded 0.80 (see Table 1). The five MA items were used as observed indicators for single-group LPA and multi-group LPA, WLE scores of mathematics interest (INTMAT), mathematics self-efficacy (MATHEFF), mathematics self-concept (SCMAT), and five plausible values of mathematics performance (PV1MATH to PV5MATH) were employed for comparisons among the three samples and derived MA profiles.

**Table 1:** PISA variables used in the study.

Variable (scale)	Number of items	Scale reliability (α)		
		FIN	KOR	USA
Mathematics anxiety	5	0.82	0.76	0.88
Mathematics performance	30^a^	0.92	0.93	0.93
Mathematics interest	4	0.90	0.91	0.91
Mathematics self-efficacy	8	0.85	0.89	0.85
Mathematics self-concept	5	0.92	0.88	0.90

*^a^Number of items each student completed for the mathematics assessment.*

### Statistical Analyses

#### Single-Group LPA

First, single LPAs with 2 to 5 solutions were conducted for the Finnish, Korean, and American samples separately to determine the number of MA profiles for these three samples. The number of MA profiles was determined on the basis of statistical criteria, the practicality, the interpretability of the extracted profiles (Bauer and Curran, 2003; Nylund et al., 2007), and the previous finding in test anxiety (von der Embse et al., 2014). We considered three statistical indices: (1) the Akaike information criterion (AIC), (2) the Bayesian information criterion (BIC), and (3) the sample-size adjusted BIC (SABIC). Lower values of the three indices indicated a better model fit and the optimal model is the one with the smallest value. However, sometimes none of the statistical criteria would arrive at the lowest value, then the best model can also be determined by the change pattern for these criteria (e.g., Morin et al., 2011), which is referred as the “elbow criterion” (Petras and Masyn, 2010). By graphing the “elbow plots” of the information criteria, we can determine the optimal solution according to the first angle of the “elbow” (Petras and Masyn, 2010). Entropy, used to assess the value and utility of the extracted profiles (range: 0–1, with bigger values indicating better classification; Petras and Masyn, 2010), the practicality of the latent groups (i.e., a sufficient number of members in each group; Collins and Lanza, 2010), and parsimony of the model (i.e., all else being equal, simpler models are preferred to more complex models; Box and Jenkins, 1976) were also considered.

#### Multi-Group LPA

Second, the response patterns of students from the three national samples on MA were examined to investigate if they are the same across the three samples, i.e., do the Finnish, Korean, and American samples have the same number of MA profiles? This step was performed by conducting and comparing a series of multigroup LPAs, including unrestricted, semi-constrained, and fully constrained MLPA models, by putting the three samples together for one data running with “country” as the grouping variable (Eid et al., 2003). For the unrestricted model, within-profile means and variances of MA and profile sizes varied across the three national samples. The measurement invariance assumption does not hold in this unrestricted model. For the semi-constrained model, profile size was still set to differ, but within-profile means and variances of MA were constrained to be equal across samples. For the fully constrained model, both profile sizes and within-profile means and variances of MA were set to be equivalent for the three national samples. Measurement invariance assumption holds in both the semi-constrained and fully constrained models. As to choose the optimal solution, we followed previous literature and chose the model with the lowest BIC value (Nylund et al., 2007; Smith et al., 2016).

All the LPA and MLPA models were analyzed in Mplus (Version 7; Muthén and Muthén, (1998–2015)) and the default robust maximum likelihood was chosen to be the estimator.

#### Validation of the Classifications

After the optimal multi-group LPA model was determined, each student in the dataset was classified to a profile according to his or her scores across the five MA indicators, and the classification was validated by conducting *post hoc* comparisons between the extracted MA profiles. Differences among the derived MA profiles were examined by testing the equality of within-group means of students’ mathematics performance and non-cognitive mathematics learning outcomes using the newly developed three-step approach of LPA, which estimates the relationships between the derived MA profiles and external variables (i.e., variables of mathematics performance and three non-cognitive outcomes in this study) by taking into account the classifying error (Bakk et al., 2013). Specifically, the AUXILIARY (BCH) function in Mplus was used (Asparouhov and Muthén, 2014). As five plausible values were used for mathematical performance, the analysis was repeated for each plausible value and the means were reported.

## Results

### Descriptive Statistics and Overall Country Level Differences

Means, standard deviations and results of mean comparisons of the PISA indices used in this study among the three samples are shown in Table 2. Students’ MA and mathematics learning outcomes all differ significantly across the Finnish, Korean, and American samples. When treated as the overall sample, Korean students demonstrated the highest level of MA, Finnish students the lowest. Korean students also had the highest scores in mathematics performance, with American students the lowest. However, the American students showed the highest level of interest, self-efficacy, and self-concept in mathematics, with Korean students the lowest. These results seemed to be confusing and inconsistent, as Finland had the lowest level of MA but not the highest mathematics performance and non-cognitive learning outcomes; Korea had the highest level of MA but showed the highest mathematics achievement, which was not consistent with the previous research findings (Hembree, 1990; Lee, 2009; OECD, 2013). American students showed a medium level of MA but the lowest performance in mathematics, and the highest level of non-cognitive mathematics learning outcomes. Korea showed the highest level of mathematics performance but the lowest level of non-cognitive learning outcomes. The inconsistency also appeared between the cognitive and non-cognitive mathematics learning outcomes.

**Table 2:** Means, SDs, and *post hoc* comparisons of mathematics anxiety and learning outcomes across the three samples.

Variable	Mean (SD)^a^			*F*	η^2^	*Post hoc* comparison
	FIN (*N* = 4746)	KOR (*N* = 2999)	USA (*N* = 3258)			
ANXMAT	−0.31 (0.99)	0.29 (0.68)	−0.10 (1.06)	371.86^∗∗∗^	0.06	KOR > USA > FIN
PVMATH	513.49 (89.04)	558.67 (96.34)	484.42 (89.20)	525.26^∗∗∗^	0.09	KOR > FIN > USA
INTMAT	−0.12 (0.96)	−0.21 (0.98)	0.12 (1.05)	49.01^∗∗∗^	0.02	USA > FIN > KOR
MATHEFF	−0.20 (0.95)	−0.31 (1.04)	0.18 (1.00)	107.88^∗∗∗^	0.04	USA > FIN > KOR
SCMAT	0.08 (1.05)	−0.38 (0.90)	0.30 (1.00)	376.36^∗∗∗^	0.06	USA > FIN > KOR

*^a^Plausible values were used for mathematics performance, while WLE scores were used for the other four variables. ^∗∗∗^p < 0.001.*

### Single-Group LPA

**FIGURE 1:**
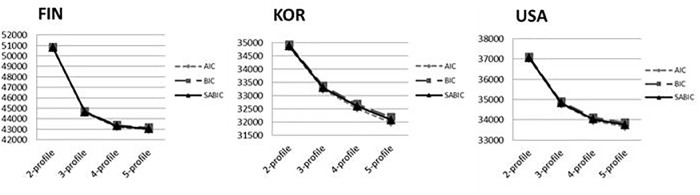
Change patterns for information criteria of different profile solutions across the three samples.

Single LPA models were determined for Finnish, Korean, and American samples separately in order to decide if the same number of MA profiles emerged in each sample. Statistical tests of model fit are presented in Figure 1 and Table 3. The three-profile model was determined based on the following considerations. First, values of AIC, BIC, and SBIC dropped dramatically at the three-profile model for all three national samples, which met Petras and Masyn’s (2010) “elbow criterion” and indicated a three- profile model was an optimal solution. Second, the values of Entropy for the three-profile model across three samples all arrived at the highest values. Third, the three-profile model was consistent with our anticipation based on the finding from a previous study on test anxiety (von der Embse et al., 2014). Thus, in terms of fit indicators, parsimony, and interpretability, a three-profile model was determined for the Finnish, Korean, and American samples in this study.

**Table 3:** Fit indices for single LPA models across the three national samples.

Sample	Model	AIC	BIC	SABIC	Entropy
FIN (*N* = 4746)	2-profile	50751.04	50854.48	50803.64	0.84
	3-profile	44568.23	44710.46	44640.55	0.99
	4-profile	43213.31	43394.33	43305.36	0.89
	5-profile	42939.44	43159.25	43051.21	0.88
KOR (*N* = 2999)	2-profile	34830.91	34927.01	34876.17	0.65
	3-profile	33229.81	33361.95	33292.04	0.99
	4-profile	32514.29	32682.46	32593.49	0.90
	5-profile	31980.14	32184.34	32076.31	0.86
USA (*N* = 3258)	2-profile	37005.77	37103.19	37052.36	0.81
	3-profile	34765.46	34899.41	34829.51	0.85
	4-profile	33945.55	34116.04	34027.07	0.84
	5-profile	33651.71	33858.73	33750.70	0.79

*AIC, Akaike information criterion; BIC, Bayesian information criterion; SABIC, sample-size adjusted Bayesian information criterion.*

### Multi-Group LPA

A multi-group LPA was carried out to test if the three-profile solution determined for any single sample separately demonstrated the same or different profile pattern across the three samples (Eid et al., 2003; Geiser et al., 2006). As shown in Table 4, the semi-constrained model had the lowest BIC value and was determined for the multi-group LPA. This indicated that the Finnish, Korean, and American samples had the same latent profile structure but the percentage of students assigned to each derived MA profile in the three national samples differed from each other.

**Table 4:** Fit indices for multi-group LPA models.

Model	AIC	BIC	SABIC
Unconstrained	145092.94	146050.01	145633.71
Semi-constrained	143806.49	144011.05	143922.07
Fully constrained	144683.25	144858.60	144782.33

After an optimal three-profile model was obtained, the derived profiles were named according to their mean scores of the five MA indicators (see Table 5). Since higher scores correspond to higher level of MA, the three profiles were named as Low MA, Mid MA, and High MA, which was consistent to von der Embse et al.’s (2014) research finding on test anxiety.

**Table 5:** Differences in profile sizes across the Finnish, Korean, and American samples.

MA profile	Sample			Mean MA score				
	FIN (*N* = 4746)	KOR (*N* = 2999)	USA (*N* = 3258)	a1	a2	a3	a4	a5
Low MA	35.29%	6.67%	25.81%	1.73	1.26	1.34	1.34	1.67
Mid MA	54.30%	71.69%	52.24%	2.81	2.08	2.18	2.18	2.86
High MA	10.41%	21.64%	21.95%	3.58	3.25	3.08	3.06	3.54

*a1 = “I often worry that it will be difficult for me in mathematics classes,” a2 = “I get very tense when I have to do mathematics homework,” a3 = “I get very nervous doing mathematics problems,” a4 = “I feel helpless when doing a mathematics problem,” a5 = “I worry that I will get poor grades in mathematics.”*

As can be seen from Table 5, differences in profile sizes appeared across the three samples for Low MA (FIN = 35.29%; KOR = 6.67%; USA = 25.81%), Mid MA (FIN = 54.30%; KOR = 71.69%; USA = 52.24%), and High MA (FIN = 10.41%; KOR = 21.64%; USA = 21.95%). Finland had the most students categorized as low in MA, Korea had the highest proportion of students with a medium level of MA, and the United States had the highest proportion of students with high level of MA.

### Validation of the Classifications

Differences among the three MA profiles were evaluated analytically by conducting several *post hoc* comparisons of the three derived MA profiles against students’ mathematics performance and non-cognitive mathematics learning outcomes (i.e., mathematics interest, mathematics self-efficacy, mathematics self-concept, and mathematics behavior) to validate the classifications from the multi-group LPA. Significant differences in the above aspects existed across the three MA profiles, as summarized in Table 6. The Low MA profile reported significantly better performance in mathematics and demonstrated more positive attitudes toward mathematics than the Mid and High MA profiles. Significant differences also existed between the Mid and High MA profile, with the Mid MA profile doing better than the High MA profile. These results were consistent with previous studies (e.g., Ashcraft and Moore, 2009), which supported the validity of classifying students into distinct MA profiles.

**Table 6:** Differences across the three MA profiles in mathematics learning: means, standard errors, and *post hoc* comparisons.

Mathematics learning outcomes	Low MA (*N* = 2716)		Mid MA (*N* = 6429)		High MA (*N* = 1858)		χ^2^	η^2^	*Post hoc* comparisons
	*M^a^*	*SE^a^*	*M^a^*	*SE^a^*	*M^a^*	*SE^a^*			
PVMATH	556.15^b^	1.69^b^	512.63^b^	1.16^b^	476.07^b^	2.12^b^	439.03^b∗∗∗^	0.07	1 > 2 > 3
INTMAT	0.43	0.03	−0.10	0.02	−0.55	0.03	279.99^∗∗∗^	0.09	1 > 2 > 3
MATHEFF	0.55	0.03	−0.21	0.01	−0.56	0.03	403.80^∗∗∗^	0.13	1 > 2 > 3
SCMAT	0.99	0.02	−0.13	0.01	−0.86	0.02	3059.63^∗∗∗^	0.36	1 > 2 > 3

*^a^Plausible values were used for mathematics performance, while WLE scores were used for the other four variables. ^b^Means of results of five repeated analysis for the five plausible values were reported. 1 = Low MA; 2 = Mid MA; 3 = High MA. ^∗∗∗^p < 0.001.*

## Discussion

The potential impact of MA on mathematical proficiency is an important issue that has received more and more attention nowadays, as research revealed that different regions of the world have different prevalence of MA (e.g., Burns, 1998; Chinn, 2009; OECD, 2015). However, estimates of the prevalence of MA in most of the studies were obtained in a single context using sum or continuous scores of MA, which might have two limitations. First, using sum or continuous of MA scores might only capture information about MA for the overall sample and probably would have obscured some important differences among different groups (Rosato and Baer, 2012), also the essential difference of MA due to the arbitrary dividing line of continuous or sum scores could not be examined. Second, research of a single cultural context would not be capable of making direct comparisons of the prevalence of MA among different countries. The present study explored the classification of MA by assigning students from Finland, Korea, and the United States into MA profiles using a multi-group LPA, trying to explore more detailed information about MA and the cultural similarities and differences of MA.

Three major points can be made from the findings in this study. First, this study was the first to investigate different profiles of MA in a large sample consisting of 11,003 15-year old students from three different countries using a latent variable mixture model, multi-group LPA. As expected, multi-group LPA identified three MA profiles (i.e., Low, Mid, and High MA profiles) for the Finnish, Korean, and American samples, which was consistent with a previous study in test anxiety (von der Embse et al., 2014). And the validity of the three MA profiles solution was supported via several *post hoc* comparisons of the three derived MA profiles against students’ mathematics learning outcomes. The knowledge of the three MA profiles could be very useful in practice when trying to categorize individuals as high or low anxious toward mathematics, especially for those who need special treatment of MA. Schools might also find this information helpful in mathematics teaching when they explore the potential sources causing students’ mathematics learning difficulty or disability, thus specific effort can be made to improve mathematics teaching and learning.

Second, by using samples of students from Finland, Korea, and the United States, this study widened research on MA from a single western context to a multicultural context combination of western and non-western contexts. Cultural similarities and differences of MA across the three samples were examined. Results indicated that Finland, Korea, and the United States shared the same latent profile structure of MA, as the same number of MA profiles consistently emerged across the three samples. It is possible that there is some universality of MA, which may not be dependent on cultural contexts. Further research would be justified on this point. Results of multi-group LPA also showed that the membership patterns differed as percentages of students classified into each MA profile were different across the Finnish, Korean, and American samples. High and Mid-MA appeared to be more prevalent among Korean students, while Low MA appeared to be more common among Finnish students. This result was consistent with Lee’s (2009) findings. It is plausible that the higher proportion of Korean students categorized as High and Mid MA stems from their greater pressure to do well on examinations, as a study by Tan and Yates (2011) revealed. Furthermore, there is an interesting finding that American students demonstrated a bit higher prevalence of High MA compared to the Korean students after the MLPA classification, while the American sample reported significantly lower levels of MA than Korean sample on the overall country level. This finding might be obscured in research using sum or continuous scores of MA of an overall sample for direct comparisons of MA across different regions or countries. The reason for American students showing a higher prevalence of High MA than Korean students may lie in their poorer mathematical attainment, as research indicated that poor mathematical attainment may lead to anxiety toward mathematics (e.g., Maloney and Beilock, 2012; Núñez-Peña and Suárez-Pellicioni, 2014). However, the mechanism of the relationship between MA and mathematics performance is much more complicated than we thought. As research indicated that there could be two causal directions between them (Carey et al., 2016); MA also leads to lower level of mathematics attitudes and they often go together and reduce mathematics involvement and achievement (Hembree, 1990). Many other cultural factors could influence mathematics performance, such as the gender and race stereotypes, instruction quality and learning time, teacher and parent expectation (e.g., Ganley and Vasilyeva, 2014; Leslie et al., 2015; Yi and Lee, 2016). This may explain why the relationship between MA and mathematics performance among different countries is not consistent, for instance, Finland demonstrated low level of MA and high mathematics achievement, while Korea had high MA but high level of mathematics performance too. More research would be needed on this point.

Thirdly, based on the two major points above, results in this study also highlighted that multi-group LPA could be a useful analytic tool for the classification and cross-culture study on MA. As a model-based probabilistic approach to classifying cases into distinct profiles and can be tested with various model fit indices, multi-group LPA identified latent profiles of MA and provided useful latent profile separation. In addition, the advanced three-step approach of LPA may also be very useful to provide validity evidence of the classification by testing the equality of within-group means of students’ mathematics performance and non-cognitive outcomes across the three MA profiles. More importantly, multi-group LPA makes it possible for direct comparisons between different countries by testing measurement invariance of different models, thus differences/similarities between different cultures can be meaningfully interpreted (Fischer and Fontaine, 2010). The application of multi-group LPA in cross-cultural research may provide an important tool to explore the cultural differences of MA in a wide context instead of a single context.

It should be noted that while the present study identified three distinct MA profiles and provided some evidence of their commonality and difference across three different regions of the world, it also had some limitations that should be carefully considered when trying to pursue the exploration further in any future studies. First, it is critical to explore cultural differences of MA with samples nationally representative to avoid any substantial sampling effects which may obscure the results of the study. Next, the reason for the cultural differences of MA is not clear so far, educational systems and curricula of different countries may also be related and are worth studying in the future (Dowker et al., 2016). Third, gender difference is a very important aspect of MA and mathematics learning as well, but we mainly focused on the cultural difference among different countries in the current study, it was hard for us to make deep research on the gender difference of MA at the mean time in this single study. Other influences, cognitive factors of individuals and pressure from parents for school achievement, may also play an important role in MA (Dowker et al., 2016). More detailed information about these factors is necessary to explain the differences of MA found in the current study and any possible future studies as well.

## Author Contributions

RH, XF, and MZ conducted the research. XF contributed to the design of the work, the acquisition, analysis and interpretation of data for the work, and drafting the manuscript. RH reviewed and revised the manuscript. MZ reviewed the manuscript and agreed to be accountable for all aspects of the work.

## Conflict of Interest Statement

The authors declare that the research was conducted in the absence of any commercial or financial relationships that could be construed as a potential conflict of interest.

## Footnotes

1 Korea refers to “South Korea” in this study.
